# “Holes” in the Jaw—A Report of Two Cases of Periapical Actinomycosis

**DOI:** 10.3390/diseases6030079

**Published:** 2018-09-06

**Authors:** Folusakin Ayoade, Ayoola Olayiwola, Ailing Li

**Affiliations:** 1 Department of Medicine, University of Miami Miller School of Medicine, Miami, FL 33136, USA; ayoola.b.olayiwola@gmail.com; 2 Dianon Labcorp, Tampa, FL 33607, USA; liailingyuhe@yahoo.com

**Keywords:** cervicofacial actinomycosis, periapical actinomycosis, osteomyelitis, osteonecrosis, penicillin therapy

## Abstract

Periapical actinomycosis is a relatively rare form of cervicofacial actinomycosis, which typically involves the periapical region with subsequent potential spread to the jaw bones. We hereby present two cases of periapical actinomycosis. Both patients presented with jaw pain and “holes” in their gum and lacked the characteristic clinical features commonly seen in cervicofacial actinomycosis such as jaw mass, draining ulcers, sinuses and fistulae. The first patient was an immunocompetent host with chronic stable medical conditions but with a rather bad dentition requiring multiple recent teeth extractions. The second patient was edentulous, had refractory multiple myeloma, was on low-dose chronic steroids and pomalidomide therapy and therefore relatively immunocompromised. Both cases of actinomycosis were diagnosed by jaw bone histopathology, which showed characteristic sulfur granules and embedded *Actinomyces*-like organisms. The two patients had excellent clinical response to six months of penicillin therapy without any need for surgical intervention. The cases remind clinicians of including actinomycosis in the differential diagnosis of periapical lesions and illustrates the possibility of achieving cure with non-surgical treatment.

## 1. Introduction

Actinomycosis is often known as the great imitator, due to its mimicry of several clinical conditions. These conditions range from bacterial, fungal, mycobacterial and parasitic infections as well as benign and malignant neoplasms [1]. Periapical actinomycosis, a subtype of cervicofacial actinomycosis, typically presents with persistent and recurrent draining fistula in the periapical region—the area around the apex of the tooth root [2]. The infection therefore has a high likelihood of spreading to the adjacent jaw bones [3]. While cervicofacial involvement is the most common overall presentation of actinomycosis, the periapical subtype is still relatively rare, though increasingly reported [2,4,5].

Cervicofacial actinomycosis typically presents as a mass at the angle of the jaw (lumpy jaw) and is commonly associated with external draining ulcers, sinuses, fistulae, and occasionally sulfur granules. The finding of “holes” or large defects of exposed jaw bone inside the mouth cavity is a relatively uncommon presentation of cervicofacial actinomycosis. We present two cases of periapical actinomycotic osteomyelitis. The first case is an immunocompetent host while the second is an immunocompromised host. Both patients presented with “holes” in their gum and lacked the characteristic clinical features commonly seen in actinomycosis. Both cases were diagnosed by histopathology and had an excellent clinical response to six months of penicillin therapy.

## 2. Case Presentation

### 2.1. Case 1

This is a 60-year-old man who presented with gum pain of one-month’s duration. His pain emanated from an area of exposed jaw bone in the left lower posterior gum. Two months earlier, he had noticed a blister in the same area, which became an abscess and was subsequently drained. He also had bad dentition that required several recent visits to his dentist. He had multiple recent teeth extractions and several artificial crowns. In addition, he had 20 pounds of weight loss as well as night sweats for the six months prior to presentation. He had no fever, neck mass or external neck draining ulcers.

His past medical history included type 2 diabetes mellitus, hypertension, hyperlipidemia and chronic obstructive pulmonary disease. He had a history of penicillin allergy (rash). He had been recently prescribed oral clindamycin for one month for his oral lesion with no improvement.

**Figure 1:**
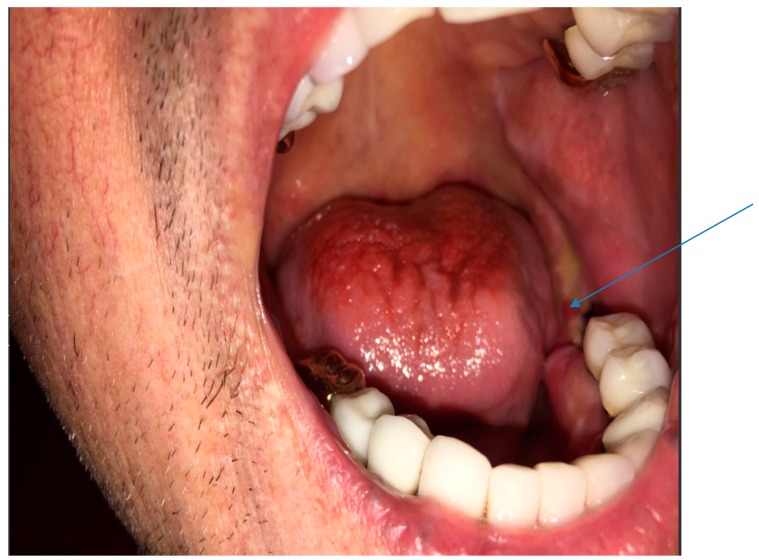
Photograph of the open mouth with arrow pointed at exposed bone around teeth #18 and 19.

His vital signs were normal. Mouth examination showed exposed bone around the root sites of teeth #18 and 19 (see arrow), with artificial crowns over several teeth in the lower jaw (Figure 1). There were no enlarged cervical lymph nodes and examination of other systems was unremarkable.

Biopsy of the left mandibular bone around the root sites of teeth #18 and 19 was obtained and sent for histopathology, aerobic and anaerobic bacterial, fungal and mycobacterial cultures. Bacterial culture grew alpha hemolytic streptococcus, *Eikenella corrodens* and *Micrococcus* spp. Fungal and mycobacterial cultures were negative.

Laboratory blood work including complete blood count, electrolytes and kidney function were completely unremarkable.

**Figure 2:**
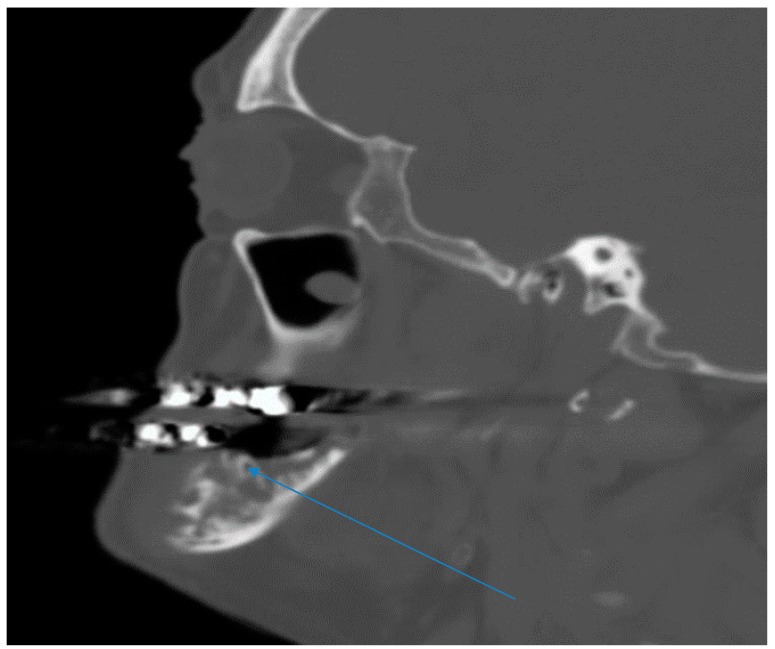
Maxillofacial computerized tomography imaging showing a lytic lesion in left ramus of the mandible with loss of bone matrix (see arrow).

Computerized tomography maxillofacial imaging showed a lytic lesion in left ramus of the mandible with loss of bone matrix (Figure 2). Chest radiograph was completely normal. Differential diagnoses considered in addition to actinomycosis included nocardiosis, tuberculosis, osteosarcoma of the mandible and endemic fungal infections.

**Figure 3:**
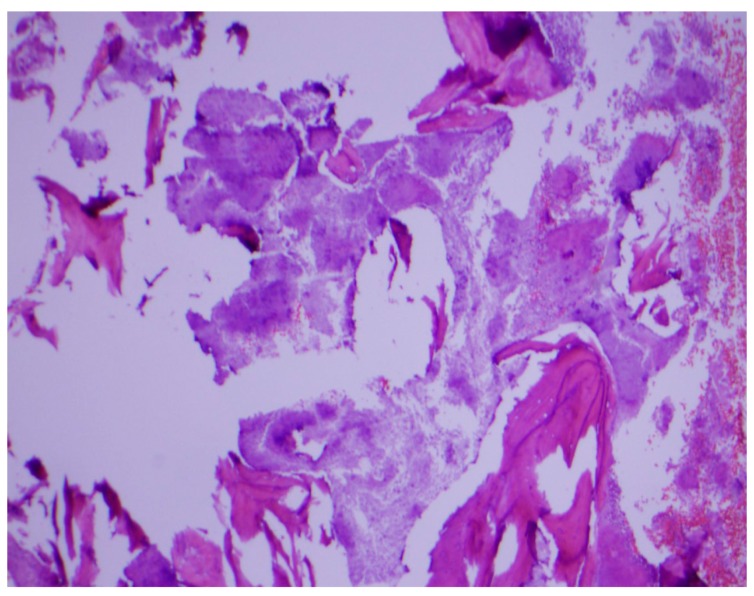
Osteonecrosis of the jaw on hematoxylin and eosin stain. Original magnification ×40.

**Figure 4:**
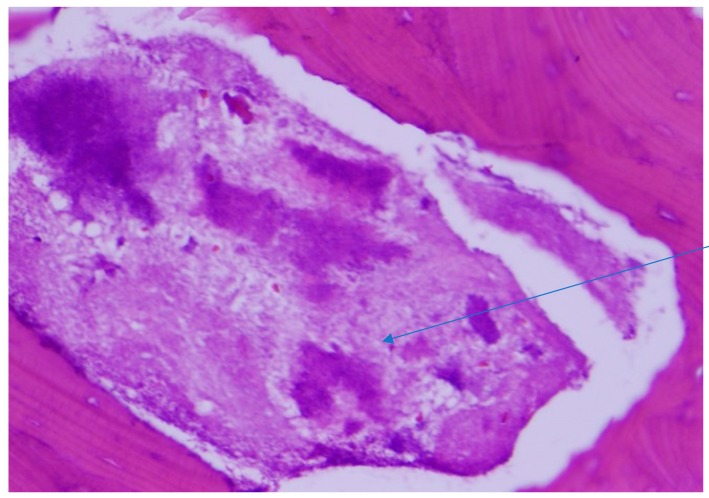
Sulfur granule (arrow) showing embedded organisms on H&E stain. Original magnification ×200.

**Figure 5:**
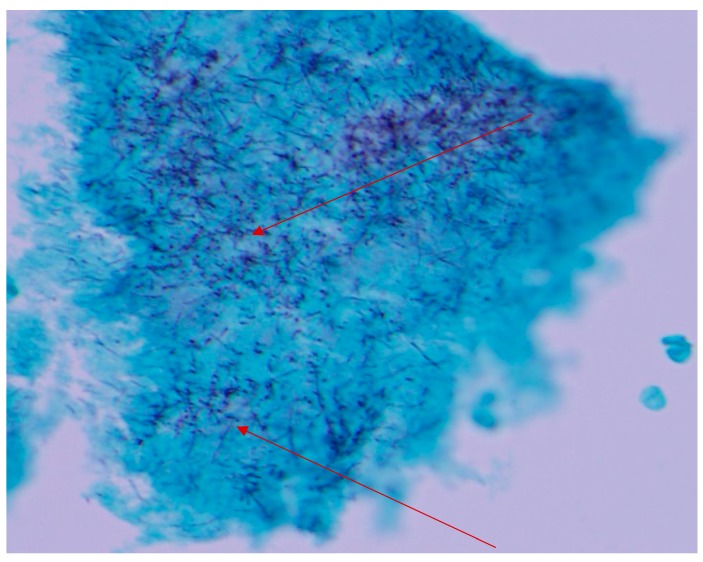
Gomori-Grocott methenamine silver stain (GMS) showing multiple branching organisms (arrows). Original magnification ×400.

Histopathological examination of the mandibular bone showed osteonecrosis, sulfur granules and embedded organisms on hematoxylin and eosin (H&E) stain (Figure 3 and Figure 4), which were better characterized on Gomori-Grocott methenamine silver stain (GMS) as multiple branching organisms (Figure 5). The official histopathology report read, “acute and chronic osteomyelitis with *Actinomyces*-like organisms”. A diagnosis of actinomycosis was made, following which the patient was desensitized and treated with intravenous penicillin G for two weeks, followed by oral penicillin VK for six months. He made a complete recovery at the end of therapy with total resolution of symptoms and closure of the exposed bone.

### 2.2. Case 2

This describes a 70-year-old woman who presented with left upper jaw pain and mastication difficulties of several weeks’ duration. She had undergone complete dental extraction three months earlier. There were no other significant complaints. Her past medical history was significant for multiple myeloma treated with pomalidomide and 20 mg weekly oral dexamethasone for nine years prior to presentation. She also had history of type 2 diabetes mellitus, hypertension and chronic kidney disease stage 3.

Vital signs were within normal limits. Her physical examination was also unremarkable except for the oral examination, which showed she was completely edentulous. In addition, she had an area of sequestrum with overlying calculus noted in the left maxilla bone corresponding to the extraction sites of teeth #11, 12 and 13. There were no surrounding lymph node enlargements or other significant examination findings.

Routine laboratory blood analysis, including complete blood count and complete metabolic profile, was unremarkable.

The piece of sequestrum was removed leaving a “hole” in the upper jaw and was subsequently sent for histopathology. The tissue sections revealed osteonecrosis, osteolytic changes with acute inflammation. The osteolytic spaces were filled with *Actinomyces*-like organisms and a few fragments of foreign material consistent with vegetables.

The clinical impression of acute osteomyelitis caused by actinomycosis was made. A possible differential that was also considered was medication-related osteonecrosis of the jaw (MRONJ), since she had been on chronic steroids and pomalidomide therapy for several years. Unfortunately, microbiologic cultures were not sent on the specimen obtained from the upper jaw.

The patient was started on a six-month regimen of penicillin VK 500 mg orally four times daily in addition to oral hygiene measures. At her clinic follow-up one month later, her symptoms had completely resolved, and the oral defect was beginning to close. At the end of her six-month therapy, she had made a complete recovery.

## 3. Discussion

The presented cases illustrate periapical actinomycotic osteomyelitis involving the jaw bones. Periapical actinomycosis is a distinct form of cervicofacial actinomycosis which may or may not be associated with the classic discharging ulcer/sinus at the angle of the jaw [2,6]. Our first patient could be considered immunocompetent while the second patient was clearly immunocompromised, given her history of refractory multiple myeloma and chronic steroid and pomalidomide use. Despite this distinction, the patients shared some similarities in their clinical presentations. The similarities included jaw or gum pain, teeth problems, jaw or gum tissue defects, absence of external draining sinuses, diagnoses made without definitive cultures, and excellent clinical response to prolonged penicillin therapy.

The cases suggest that unexplained gum or jaw problems, especially in the setting of “holes in the gum”, should raise the possibility of cervicofacial actinomycosis. The typical mass at the angle of the jaw (lumpy jaw), draining sinuses with granules from discharging ulcers, abscess formation, fistulae and fibrous tissue formation are not always present, as typified by our cases. In a similar fashion, Kim et al. reported a presentation of actinomycosis mimicking periodontitis without the other classic features associated with cervicofacial actinomycosis [7].

*Actinomyces* are best described as fastidious, branching, anaerobic to microaerophilic gram-positive bacteria that typically cause acute, subacute or chronic infection in different organ systems. The infection can cross several tissue planes and mimic several other conditions, including malignancy [8]. By crossing tissue planes, the ensuing tissue destruction can result in devastating outcomes, including massive hemorrhage, secondary infection, increased intracranial pressure and septic emboli [6]. The predisposition to cervicofacial actinomycosis in relationship to poor dentition is not far-fetched given the established knowledge that *Actinomyces* are normal mouth flora [9]. They are also more prevalent in dental plaques, periodontal pockets, gingival crevices and tonsillar crypts. Poor dentition was potentially present in both of our cases and was likely a factor in causing periapical actinomycosis in both patients. Tooth extraction or significant gingival manipulation can also be associated with actinomycosis since disruption of the oral mucosa potentially exposes deeper gingival and periodontal tissues to the *Actinomyces*-rich oral flora [9,10]. While the mandible is the most commonly involved structure in cervicofacial actinomycosis, involvement of the maxilla is typically rare, accounting for only 5.7% in one review [11,12]. Nevertheless, the maxilla is more commonly involved than the mandible in periapical actinomycosis [4].

Bacterial culture did not grow *Actinomyces* in our first patient while culture information was not available for our second case. Notwithstanding, given the very fastidious nature of *Actinomyces*, pathology is a more reliable way for diagnosis, as typified by our first case. Unreported recent antibiotic use, anaerobic requirements and the presence of other competing pathogens often impede the yield of positive *Actinomyces* culture [13]. Even when bacterial culture is positive, it is often polymicrobial [14,15]. The anaerobic milieu and synergistic contribution between the other mouth bacteria and *Actinomyces* has been potentially implicated in the tissue destruction seen in actinomycosis [16].

Medication-related osteonecrosis of the jaw (MRONJ) from chronic steroids and pomalidomide use was a diagnostic consideration for our second case. Non-infectious osteonecrosis has been associated with the use of several chronic medications, including intravenous high potency bisphosphonates, denosumab [17], corticosteroids [18], and combination of corticosteroids with taxanes and denosumab [19]. The possible association between actinomycotic osteomyelitis and MRONJ has been previously described and this could also have played a role in the development of osteomyelitis in our second patient [20,21,22].

The contribution of host factors in the predisposition to actinomycosis has been previously published [23,24]. In addition to the immunocompromised state demonstrated in our second patient, other potential host factors present in both patients include age (20 to 60) and diabetes mellitus. Even though our second patient was only taking 20 mg dexamethasone weekly, the cumulative dose and duration of steroid therapy were likely important in contributing to impaired immunity and subsequent increased infection risk [25,26].

Pomalidomide is a thalidomide analogue with antineoplastic, anti-angiogenetic and immunomodulatory properties [27]. Like thalidomide, it is also considered teratogenic. It has a role, in combination with low-dose dexamethasone, in the treatment of multiple myeloma [28,29]. There is evidence to suggest that prolonged therapy with pomalidomide may impair immunity [30].

Our second patient was edentulous. While edentulism is theoretically expected to be associated with less oral infection risk, the negative impact on oral and general health is enormous [31]. The role of complete edentulism as a possible risk factor for actinomycosis has not been well studied, even though limited association has been suggested for mycoplasma and a few other infections [32,33]. Further studies to explore such possible association is recommended.

Both our patients had osteomyelitis in association with actinomycosis and received prolonged therapy for six months with penicillin, which is considered the drug of choice [11,34]. There are, however, other potential treatment options for actinomycosis, including clindamycin, erythromycin, tetracyclines, lincomycin, vancomycin and chloramphenicol [35,36]. Success with shorter treatment options have also been described but will often require surgical debridement [11,37]. Fluoroquinolones, metronidazole and aminoglycosides are considered poor choices, but there is generally good clinical experience with tetracycline and doxycycline even though some regard them as less preferred options [34]. Additionally, some isolates, including *Actinomyces europaeus* and *Actinomyces graevenitzii*, are generally resistant to cephalosporins [23,37]. Since almost all isolates are penicillin-sensitive, susceptibility results are seldom pursued. Even though there are several species of Actinomyces, about 70% of all cervical actinomycosis are caused by *A. israelii* and *A. gerencseriae* [37]. Finally, when significant tissue necrosis is present, surgical debridement of necrotic tissue is often required for a successful outcome, in addition to appropriate antibiotic therapy [23,38]. Neither of our cases required surgical debridement.

In summary, jaw pain associated with “holes” in the gum or longstanding gum tissue defects should raise suspicion for periapical actinomycosis in the right clinical setting. Making the correct diagnosis may prevent unnecessary work-up and treatment for malignancy which is often a common misdiagnosis in this scenario. The characteristic draining sinus, chronic skin ulcers and fistulae are not always present. Histopathology is often the mainstay of diagnosis and prolonged penicillin therapy for at least six months is the preferred therapy. Surgical debridement is not always necessary as typified by our cases.
